# Cronkhite–Canada Syndrome With Multiple Mesenteric Lymphadenopathy: A Case Report

**DOI:** 10.1002/jgh3.70309

**Published:** 2025-12-09

**Authors:** Takashi Nishino, Chikamasa Ichita, Akiko Sasaki, Chihiro Sumida

**Affiliations:** ^1^ Gastroenterology Medicine Center Shonan Kamakura General Hospital Kamakura Kanagawa Japan

**Keywords:** autoimmune mechanism, Cronkhite–Canada syndrome, mesenteric lymphadenopathy, polyposis

## Abstract

Cronkhite–Canada syndrome (CCS) is a rare nonhereditary disorder characterized by multiple gastrointestinal polyps and ectodermal changes. The mortality rate can reach up to 50% in patients with delayed diagnosis or inadequate treatment. A 78‐year‐old Japanese woman presented with diarrhea as the primary complaint. Her clinical presentation included diarrhea, dysgeusia, anorexia, and weight loss. Physical examination revealed alopecia, nail atrophy, and hyperpigmentation. Abdominal computed tomography (CT) revealed multiple enlarged mesenteric lymph nodes, whereas endoscopic examination showed numerous hyperplastic polyps extending from the stomach to the colon. Following the diagnosis of CCS, the patient was treated with prednisolone (30 mg/day). Abdominal CT imaging one month later showed a reduction in the mesenteric lymph node size. Although it is uncommon, mesenteric lymphadenopathy can appear in CCS and may regress with corticosteroid therapy.

**Abbreviations**
CCS: Cronkhite–Canada syndrome
CT: computed tomography

## 1 Introduction

Cronkhite–Canada syndrome (CCS) is a rare nonhereditary disorder characterized by chronic diarrhea, malnutrition, alopecia, nail atrophy, and skin hyperpigmentation. First described in 1955 [1], over 500 cases have been documented, with more than 75% originating from Japan [2]. Although the exact cause of CCS remains unclear, the positive outcomes of immunosuppressive therapies, including glucocorticoids, azathioprine, anti‐tumor necrosis factor antibodies, and cyclosporine, indicate an autoimmune mechanism [3]. Furthermore, patients with CCS have a significantly higher risk of developing gastrointestinal malignancies than the general population, warranting vigilant surveillance [2]. The mortality of CCS can be up to 50% [4], in cases of delayed or inadequate treatment. Early diagnosis and treatment are essential for improving outcomes and increasing remission rates.

While there have been reports documenting the endoscopic and histopathological changes before and after treatment for CCS [5], there is a lack of literature reporting the presence of abdominal lymphadenopathy on computed tomography (CT) imaging. We report a case of CCS in which mesenteric lymphadenopathy was observed on CT imaging, which subsequently resolved following treatment.

## 2 Case Report

**FIGURE 1:**
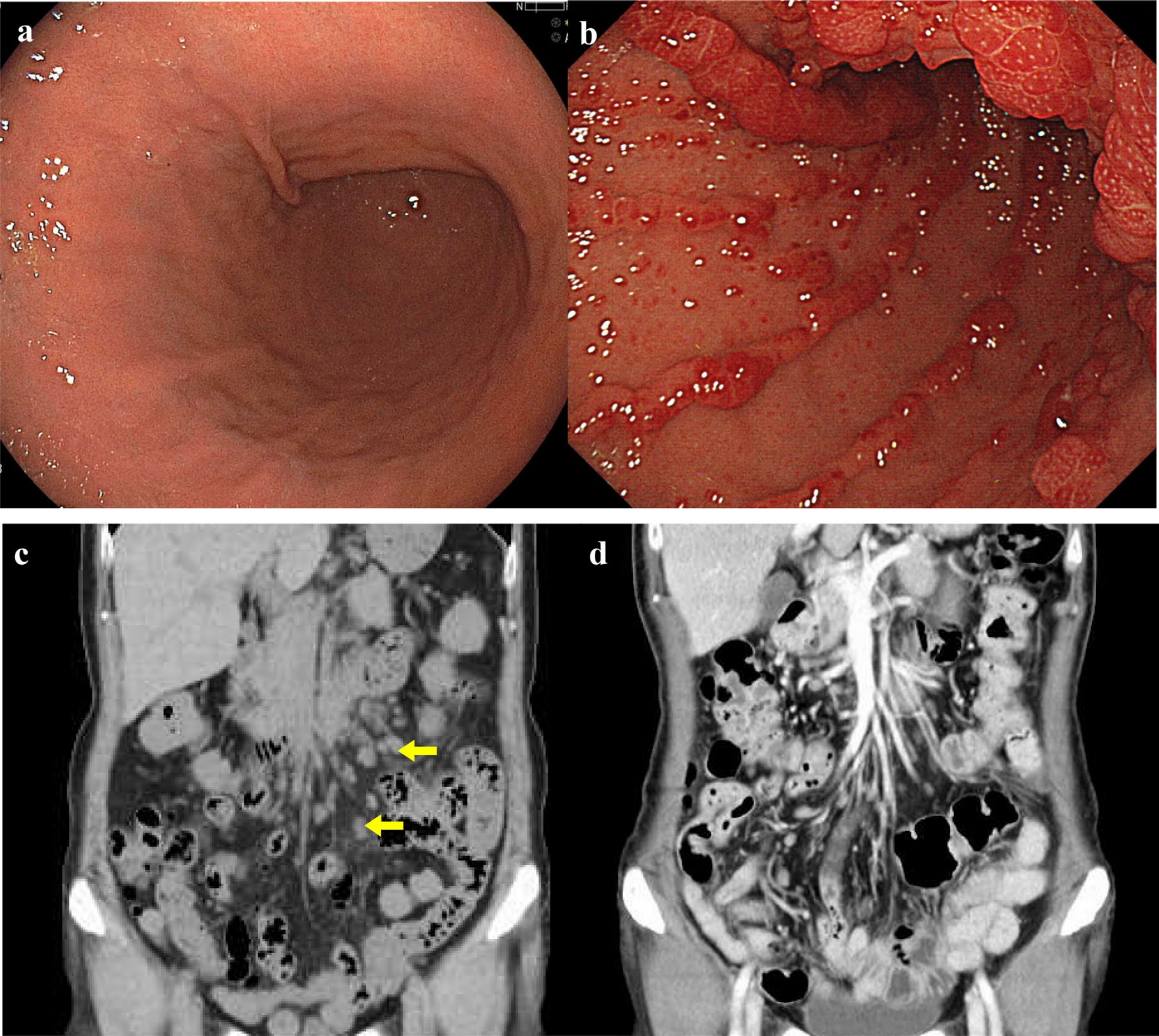
(a) Upper gastrointestinal endoscopy findings 1 year before visit. (b) Upper gastrointestinal endoscopy findings after consultation by referral. (c) CT scan of the abdomen at the time of referral showed multiple enlarged mesenteric lymph nodes. (d) A month after treatment, the mesenteric lymph node swelling had disappeared.

A 78‐year‐old Japanese woman presented with diarrhea. One year earlier, upper gastrointestinal endoscopy revealed non‐atrophic mucosa without *Helicobacter pylori* infection or gastric fundic gland polyps (Figure 1a). The patient had no history of smoking or other relevant family history. Six weeks before presenting, she experienced dysgeusia, watery diarrhea up to 10 times daily, and a weight loss of 6 kg (13.6% of initial body weight) over 3 months. The previous physician had performed an abdominal CT scan, which revealed multiple mesenteric lymphadenopathies, prompting the referral to our institution.

On examination at our institute, the patient was found to exhibit hyperpigmentation of the lower lip and palms, nail bed atrophy, hair loss, and pitting edema in both lower legs. Laboratory findings revealed hypoalbuminemia, hypozincemia, an elevated ESR, and increased soluble interleukin receptor levels (Table 1). Abdominal CT tomography confirmed multiple enlarged mesenteric lymph nodes (Figure 1c). Considering the risk of lymph node metastasis from gastrointestinal cancer or lymphoma, upper and lower gastrointestinal endoscopies were performed. Upper endoscopy revealed diffuse, sessile, hemispherical polyps measuring 2–5 mm in diameter with erythematous surfaces throughout the stomach (Figure 1b). Lower endoscopy revealed multiple strawberry‐like, nodular, smooth, reddish, engorged, nonpedunculated polyps with pit openings, 3–5 mm in size, diffusely spread throughout the colon. No evidence of malignancy was observed. Random biopsies performed during upper and lower gastrointestinal endoscopy revealed no neoplastic lesions but demonstrated chronic inflammation and mucosal hyperplasia.

**TABLE 1:** Laboratory results on presentation.

Parameters	Findings
White blood cell count (/μl)	4200
Hemoglobin (g/dl)	12.1
Mean corpuscular volume (fl)	101.5
Platelet count (×10^3^/μl)	223
Total protein (g/dl)	5.9
Albumin (g/dl)	3.1
Zinc (μg/dl)	74.8
C‐reactive protein (mg/dl)	0.23
Blood urea nitrogen (mg/dl)	8.1
Creatinine (mg/dl)	0.46
Soluble interleukin‐2 receptor (U/ml)	1849
Erythrocyte sedimentation rate at 1 h (mm)	26
T‐SPOT.TB test	Negative

The patient was diagnosed with Cronkhite–Canada syndrome based on the diagnostic criteria established by the Ministry of Health, Labour and Welfare of Japan, having fulfilled all three major clinical features: (1) nonhereditary multiple non‐neoplastic gastrointestinal polyps; (2) chronic diarrhea; and (3) characteristic ectodermal findings, including hair loss, nail atrophy, and skin pigmentation. The disease was classified as severe due to frequent bowel movements, low serum albumin level, pitting edema, weight loss of > 5 kg in 6 months, skin findings, and dysgeusia. Treatment with oral prednisolone (30 mg/day) was initiated, resulting in the resolution of diarrhea within 4 weeks and improvement in hair loss within 5 weeks. A follow‐up CT scan after 1 month showed a reduction in the size of the mesenteric lymph nodes (Figure 1d). The patient was referred to a hematologist for further evaluation of mesenteric lymphadenopathy. The enlargement was limited to mesenteric nodes that drain the gastrointestinal tract. Upper and lower endoscopy showed no evidence of primary gastrointestinal lymphoma. The soluble interleukin‐2 receptor was mildly elevated but could be explained by CCS‐related inflammation. Bone‐marrow biopsies performed on two separate occasions revealed no fibrosis, dysplasia, or malignant infiltration. Based on these findings, the hematology team judged malignant lymphoma unlikely and recommended lymph‐node biopsy only if re‐enlargement occurred during steroid tapering.

## 3 Discussion

No previous reports have documented mesenteric lymphadenopathy in patients with CCS. In the present case, primary malignancy and lymphoma were essentially ruled out. The resolution of mesenteric lymphadenopathy after prednisolone therapy suggests that lymphadenopathy is associated with CCS.

The mechanism underlying lymphadenopathy in CCS is unknown. Common causes of mesenteric lymphadenopathy include lymph node metastasis of malignant tumors, malignant lymphoma, gastrointestinal infections, and reactive enlargement due to systemic diseases such as IgG4‐related disease (IgG4‐RD), Castleman's disease, and inflammatory bowel disease [6, 7]. Previous reports have suggested that autoimmune mechanisms play a role in CCS, as evidenced by its responsiveness to anti‐inflammatory therapy, histopathological evidence of IgG4‐positive cells, and frequent positivity to antinuclear antibodies [2, 3]. In this case, a significant improvement in lymphadenopathy was observed after steroid therapy. Malignancy was also excluded based on imaging studies, including gastrointestinal endoscopy. The resolution of lymphadenopathy suggests that its cause was not carcinoma metastasis but may be associated with the inflammatory mechanisms of CCS. However, neither serum IgG4 levels nor the presence of IgG4‐positive plasma cells in tissue samples was assessed in this case, and further research is required to elucidate the underlying mechanisms.

Patients with CCS may present with adenomatous polyps or concurrent malignant tumors at diagnosis, with reports indicating up to a 15% incidence of gastrointestinal malignancies [5, 8]. Therefore, in cases of mesenteric lymphadenopathy, the evaluation of lymph node metastasis from malignant tumors is essential. In our case, routine endoscopic examinations, including upper and lower gastrointestinal endoscopies, performed within the past year did not identify any malignant tumors. Malignant neoplasms were not strongly suspected in the absence of endoscopic or histopathological evidence at the time of diagnosis.

Delayed diagnosis is a potential factor for poor prognosis, because CCS is a rare condition with often unrecognized clinical symptoms, leading to frequent misdiagnosis. Recent advancements in anti‐inflammatory treatments have improved CCS [9]. However, the risk of severe malnutrition remains high without an early diagnosis or timely initiation of therapy [4]. Early diagnosis is critical, requiring awareness of the characteristic clinical symptoms and endoscopic findings, and consideration of CCS as a differential diagnosis in cases presenting with mesenteric lymphadenopathy.

A limitation of this report is that follow‐up imaging after steroid tapering was unavailable; therefore, late re‐enlargement of the mesenteric lymph nodes or an underlying lymphoma cannot be completely ruled out.

## 4 Conclusion

This report describes a rare case of multiple mesenteric lymphadenopathies in a patient with CCS. Although it is uncommon, mesenteric lymphadenopathy can appear in CCS and may regress with corticosteroid therapy.

## Funding

The authors have nothing to report.

## Ethics Statement

The procedure was performed in accordance with the ethical standards of the Declaration of Helsinki and its later amendments.

## Consent

Informed consent was obtained from this patient.

## Conflicts of Interest

The authors declare no conflicts of interest.

## Data Availability

Data sharing is not applicable to this article as no new data were created or analyzed in this study.
